# Case Report: A rare case of external iliac artery rupture and sigmoid fistula following multimodal treatment for cervical cancer

**DOI:** 10.3389/fonc.2026.1708443

**Published:** 2026-02-02

**Authors:** Xu Cheng, Jiantao Wang, Huanzhong Wang, Haotian Song, Guoping Zhao, Hongzhi Wang

**Affiliations:** 1 Hefei Cancer Hospital of CAS, Institute of Health and Medical Technology, Hefei Institutes of Physical Science, Chinese Academy of Sciences (CAS), Hefei, Anhui, China; 2 Science Island Branch, Graduate School of University of Science and Technology of China, Hefei, Anhui, China; 3 High Magnetic Field Laboratory, Hefei Institutes of Physical Science, Chinese Academy of Sciences, Hefei, China

**Keywords:** cervical cancer, complication, external iliac artery rupture, multidisciplinary management, postoperative radiotherapy, sigmoid fistula

## Abstract

We report a 58-year-old woman with cervical squamous cell carcinoma who developed an early-onset left external iliac artery rupture followed by a sigmoid fistula after multimodal treatment. Notably, key baseline tumor parameters-including maximal tumor diameter and stromal invasion depth-were not documented preoperatively because PET-CT was not performed and the original MRI/CT datasets from the outside hospital were unavailable. This incomplete staging likely contributed to underestimation of disease extent and to selection of a non-guideline-concordant primary radical hysterectomy, although postoperative pathology ultimately confirmed FIGO 2018 stage IIIC2 disease with extensive nodal metastases. The patient subsequently received adjuvant pelvic external-beam radiotherapy and vaginal cuff high-dose-rate brachytherapy. One month after completing radiotherapy, she presented with acute hematochezia and hemorrhagic shock. Angiography revealed active extravasation from the left external iliac artery adjacent to a postoperative lymphocele, and a covered stent was deployed with temporary hemostasis. Despite intensive antimicrobial and supportive therapy, she later developed a sigmoid fistula, pelvic abscess, recurrent bleeding, and persistent sepsis, culminating in fatal deterioration. The arterial rupture was considered to be multifactorial, with potential contributions from extensive lymphadenectomy, lymphocele formation, infection, and radiation-related tissue fragility. This case underscores the critical importance of comprehensive preoperative assessment and guideline-based primary treatment selection in cervical cancer, as inappropriate initial management may predispose patients to severe lymphatic, infectious, and vascular complications. Early multidisciplinary surveillance and timely intervention may be essential to prevent catastrophic outcomes. In gynecologic oncology, the combination of early-onset iliac arterial rupture and subsequent sigmoid fistula shortly after postoperative radiotherapy remains exceedingly rare, and to our knowledge has not been previously reported in a patient with unrecognized FIGO IIIC2 disease at initial treatment.

## Introduction

Cervical cancer remains the fourth most common malignancy among women worldwide. In 2022, approximately 660,000 new cases and 350,000 deaths were attributed to this disease, accounting for 6.9% of all cancer-related mortality (1).Treatment strategies are stage-dependent and should be individualized according to histology, tumor size, patient comorbidities, fertility considerations, and available resources. Standard modalities include surgery, radiotherapy, chemotherapy, and, increasingly, systemic therapies such as immunotherapy (2).

Although multimodal treatment improves oncologic control in appropriately selected patients, it also increases treatment-related morbidity. Severe complications—including major vascular rupture, enteric fistula formation, and deep pelvic infection—are uncommon but potentially fatal and can substantially compromise quality of life and survival (3, 4). After radical hysterectomy, postoperative risk stratification has traditionally relied on the Gynecologic Oncology Group (GOG) intermediate-risk and high-risk frameworks. The Sedlis criteria (GOG 92) define intermediate-risk features (deep stromal invasion, lymphovascular space invasion, and tumor diameter >4 cm) that are associated with increased pelvic recurrence and inform the use of adjuvant pelvic radiotherapy (5, 6). In contrast, the Peters criteria (GOG 109) emphasize high-risk features—pelvic lymph node metastasis, parametrial involvement, or positive margins—which strongly support adjuvant concurrent chemoradiotherapy (7).However, the clinical utility of these historical criteria depends on accurate baseline assessment of tumor size, stromal invasion depth, and nodal status. When key preoperative tumor characteristics are unavailable, treatment selection may be based on incomplete staging, increasing the risk of inappropriate initial management and avoidable downstream multimodal toxicity.

Contemporary guidelines provide clearer direction for primary treatment selection. According to the NCCN Guidelines (Version 2.2026) (8) and the ESGO/ESTRO/ESP 2023 recommendations, patients with suspected or confirmed pelvic or para-aortic nodal metastasis (FIGO 2018 stage IIIC1/IIIC2) should generally receive definitive concurrent chemoradiation as initial therapy. Primary radical hysterectomy is not recommended in such cases because it offers no survival benefit and increases surgical morbidity, often necessitating additional adjuvant treatment. Therefore, comprehensive preoperative staging—including high-quality pelvic MRI and functional imaging when indicated, with access to complete imaging datasets—is essential to ensure guideline-concordant treatment and to minimize preventable complications.

When adjuvant radiotherapy is indicated, the benefit in reducing pelvic recurrence is well established. In GOG 92, adjuvant pelvic radiotherapy initiated within 4–6 weeks after surgery reduced the risk of pelvic recurrence by approximately 39%, with severe or life-threatening toxicity reported in 6% of patients compared with 2% in the observation arm (9). Similarly, GOG 109 demonstrated improved local control with postoperative concurrent chemoradiotherapy, although grade 4 toxicity occurred more frequently than with radiotherapy alone (17% vs. 4%) (7). These pivotal trials underscore that postoperative irradiation is effective when appropriately indicated but is accompanied by clinically meaningful toxicity, particularly in patients already burdened by extensive surgery and postoperative complications.

Radiotherapy can also induce vascular injury through endothelial dysfunction, chronic inflammation, fibrosis, and progressive vessel wall fragility (10). Classically, radiation-induced vasculopathy presents as a delayed complication—often manifesting as aneurysm or pseudoaneurysm formation and, rarely, rupture—most frequently reported in the intracranial or carotid circulation with an average latency of 10–20 years (11, 12). Available evidence suggests a dose-dependent process mediated by oxidative stress and pro-inflammatory cytokines, with risk influenced by cumulative dose, patient-related factors, and irradiation of critical vascular territories (13, 14). In gynecologic oncology, however, vascular catastrophes after pelvic irradiation are exceedingly rare, and published cases typically involve heavily pretreated or recurrent malignancies in which surgery, re-irradiation, stent placement, and infection likely act synergistically (15–19). Reports of early bowel–vascular fistulae exist, but they remain exceptional and are often associated with severe local contamination and prior pelvic interventions (17, 20). Importantly, systematic studies evaluating early-onset arterial rupture in the postoperative adjuvant setting are lacking.

Here, we describe an unusually early sequence of catastrophic complications—left external iliac artery rupture followed by sigmoid fistula—occurring within one month after completion of postoperative radiotherapy for cervical cancer. This case is distinctive not only for its extreme early onset compared with the typical decade-long latency reported for radiation-associated vasculopathy, but also for its occurrence in a patient who underwent a non–guideline-concordant primary radical hysterectomy because essential baseline imaging parameters were unavailable and nodal disease (ultimately FIGO 2018 stage IIIC2) was not recognized at initial treatment. We highlight the likely multifactorial pathogenesis involving surgical trauma, lymphocele formation, infection, and radiation-related tissue fragility, and we emphasize the clinical importance of comprehensive staging, guideline-based primary treatment selection, and vigilant multidisciplinary surveillance to prevent catastrophic events in cervical cancer management.

## Case description

A 58-year-old woman presented to an outside hospital with mild spontaneous vaginal bleeding for 3 months. Pelvic examination revealed a friable cervical mass extending to the upper two-thirds of the vagina, without obvious parametrial thickening. Pelvic MRI and chest–abdominal CT reportedly demonstrated a cervical lesion confined to the cervix and upper vagina, with no radiologic evidence of parametrial invasion or nodal/distant metastasis. Based on these findings, the disease was clinically staged as FIGO 2018 stage IIA. However, PET-CT was not performed, and the original MRI/CT datasets were unavailable for independent review. Consequently, essential baseline tumor characteristics—particularly maximal tumor diameter and stromal invasion depth—could not be verified and remain unknown. Cervical biopsy confirmed invasive poorly differentiated squamous cell carcinoma. On admission, vital signs were stable, and physical examination was otherwise unremarkable.

The patient subsequently underwent an open radical hysterectomy (Querleu–Morrow type C1) with bilateral salpingo-oophorectomy, pelvic lymph node dissection, para-aortic lymph node dissection, and presacral lymph node dissection at the same institution. The surgical decision was made based on the available preoperative evaluation (pelvic MRI and chest–abdominal CT only). Final histopathology revealed lymphovascular space invasion, perineural invasion, and metastases in 12 of 20 resected lymph nodes, consistent with FIGO 2018 stage IIIC2 disease. Given the extent of nodal involvement, definitive concurrent chemoradiation would have been the guideline-concordant primary treatment; therefore, this initial surgical approach likely increased postoperative morbidity and the subsequent need for multimodal therapy.

**Figure 1:**
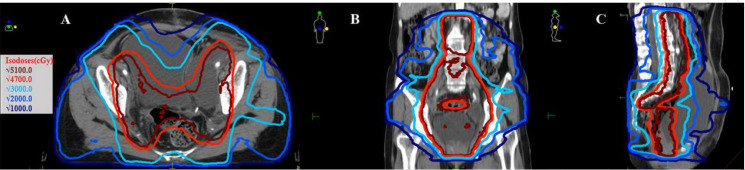
Radiotherapy dose distribution and target coverage. **(A)** Axial view of the pelvic radiotherapy plan showing isodose lines and target volume coverage. **(B)** Coronal view demonstrating dose distribution across the pelvis. **(C)** Sagittal view illustrating isodose distribution and target coverage (prescribed dose: 50.4 Gy in 28 fractions). Gy, Gray.

One month after surgery, adjuvant concurrent chemoradiation was initiated because of postoperative high-risk features. External beam radiotherapy (EBRT) was delivered to the pelvis at a total dose of 50.4 Gy in 28 fractions (**Figure 1**), with weekly cisplatin (40 mg). Vaginal cuff high-dose-rate brachytherapy was subsequently administered (18 Gy in 3 fractions, once to twice per week). The treatment course was completed without acute grade ≥3 toxicity. During EBRT, the patient developed progressive left-leg pain. Pelvic ultrasound and CT demonstrated a persistent postoperative lymphocele adjacent to the left external iliac vessels, suggesting ongoing lymphatic leakage and local inflammatory change within the irradiated field. At 3 months post-surgery, ultrasound-guided drainage of the lymphocele was performed with symptomatic relief, and cytology was negative for malignant cells.

**Figure 2:**
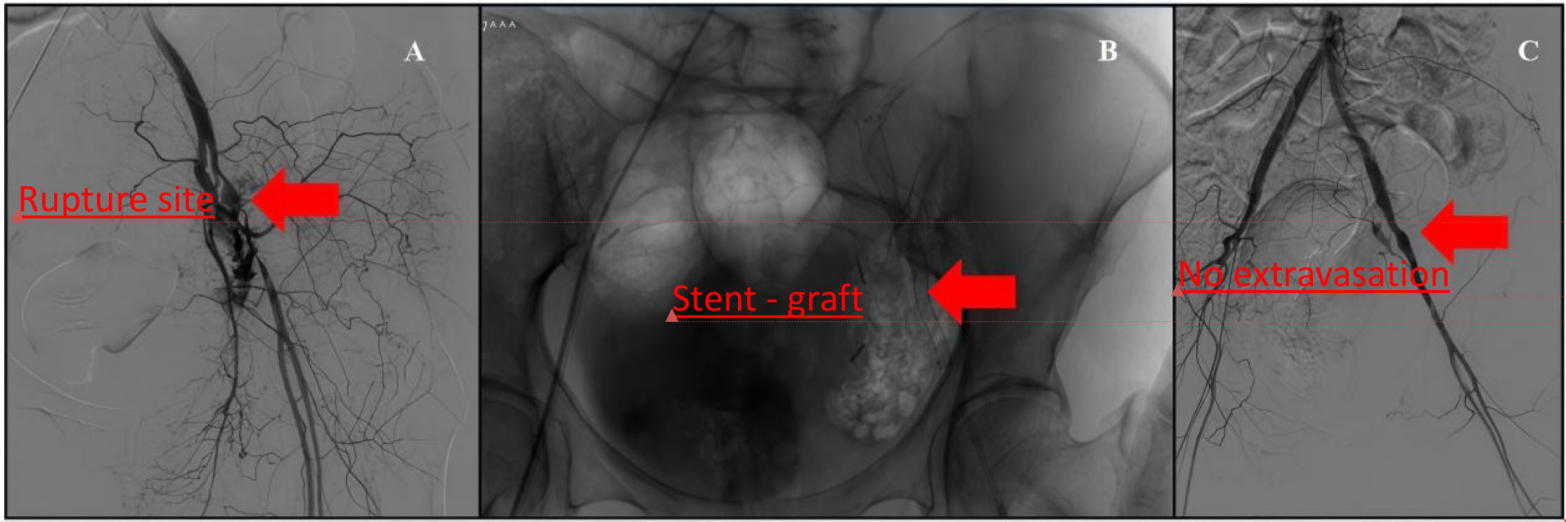
Emergency digital subtraction angiography (DSA) and endovascular management of left external iliac artery rupture. **(A)** Pre-intervention DSA demonstrating active contrast extravasation from the left external iliac artery (arrow), consistent with arterial rupture. **(B)** Fluoroscopic image showing deployment of a covered stent-graft across the rupture site (arrow). **(C)** Completion angiography after stent-graft placement demonstrating restoration of luminal patency and complete cessation of contrast extravasation (arrow).

At 4 months post-surgery (approximately 1 month after completion of radiotherapy), the patient presented with sudden-onset hematochezia without significant abdominal pain or distension. Digital rectal examination revealed a large amount of fresh blood and clots. Differential diagnoses at that time included radiation proctitis, colonic ulceration, tumor-related bleeding, and vascular injury. Digital subtraction angiography (DSA) demonstrated active contrast extravasation from the left external iliac artery adjacent to the postoperative lymphocele (**Figure 2**), and the patient rapidly developed hemorrhagic shock. Emergency endovascular deployment of a covered stent across the rupture site achieved immediate hemostasis and hemodynamic stabilization.

**Figure 3:**
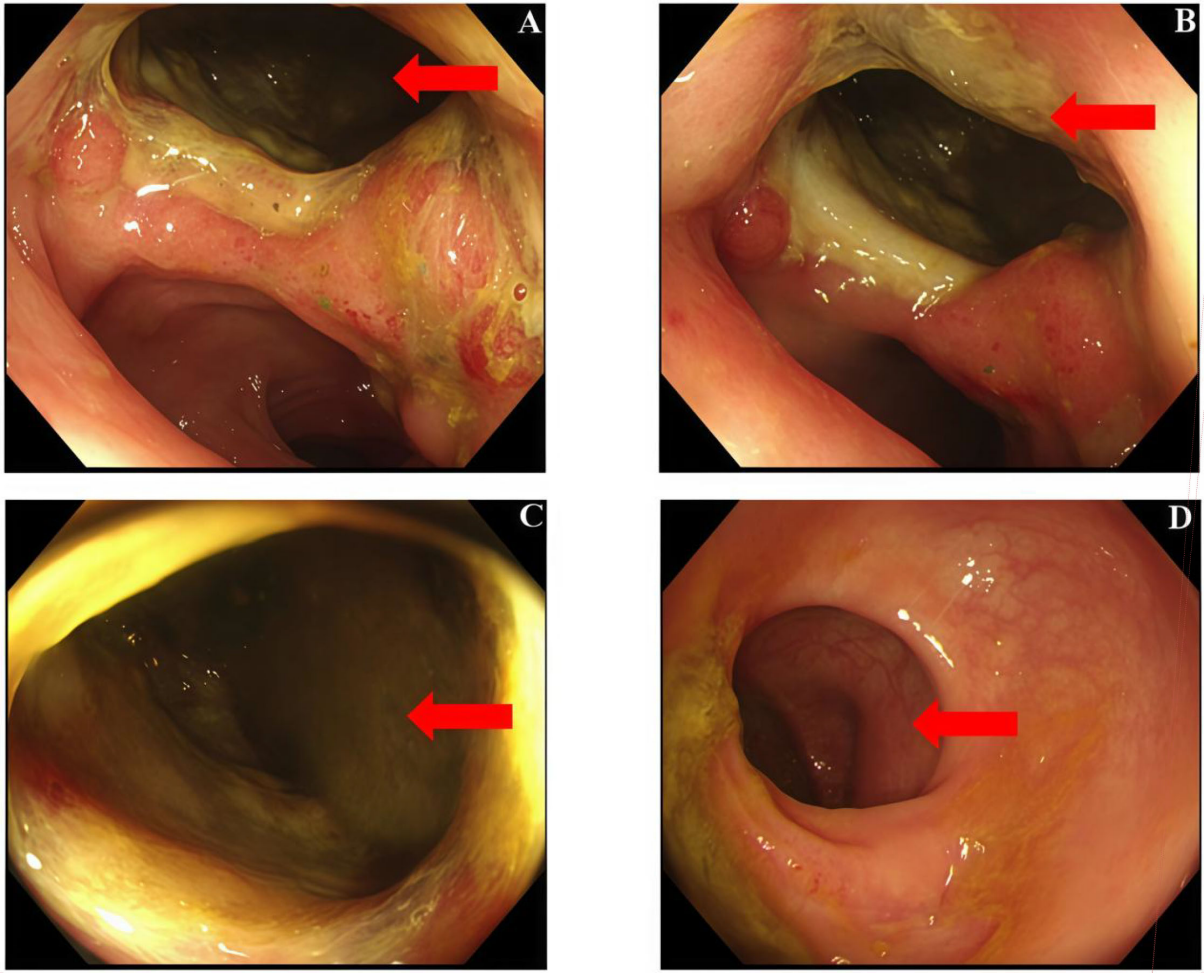
Colonoscopic findings confirming a sigmoid colonic fistula. **(A, B)** Representative colonoscopic views showing an abnormal fistulous tract in the sigmoid colon with surrounding inflamed and exudative mucosa (arrows). **(C)** Fistula opening in the sigmoid colon (arrow). **(D)** Rectal lumen showing no visible fistula at this level (arrow indicates the rectal lumen).

Despite initial bleeding control, the patient deteriorated over the ensuing weeks. At 5 months post-surgery, she developed recurrent intermittent hematochezia, persistent fever, and progressive malnutrition. Colonoscopy confirmed a sigmoid colon fistula (**Figure 3**). Contrast-enhanced CT revealed a gas-containing cystic lesion adjacent to the left iliac vessels, consistent with an infected pelvic collection, with associated hematoma and suspected invasion/adhesion to the bladder wall. Serial laboratory evaluation showed severe anemia, hypoalbuminemia, and electrolyte disturbances, consistent with ongoing blood loss, systemic inflammation, and poor nutritional reserve. During hospitalization, she required repeated blood transfusions, albumin supplementation, broad-spectrum intravenous antibiotics, drainage and supportive care, and intensive nutritional therapy.

**Figure 4:**
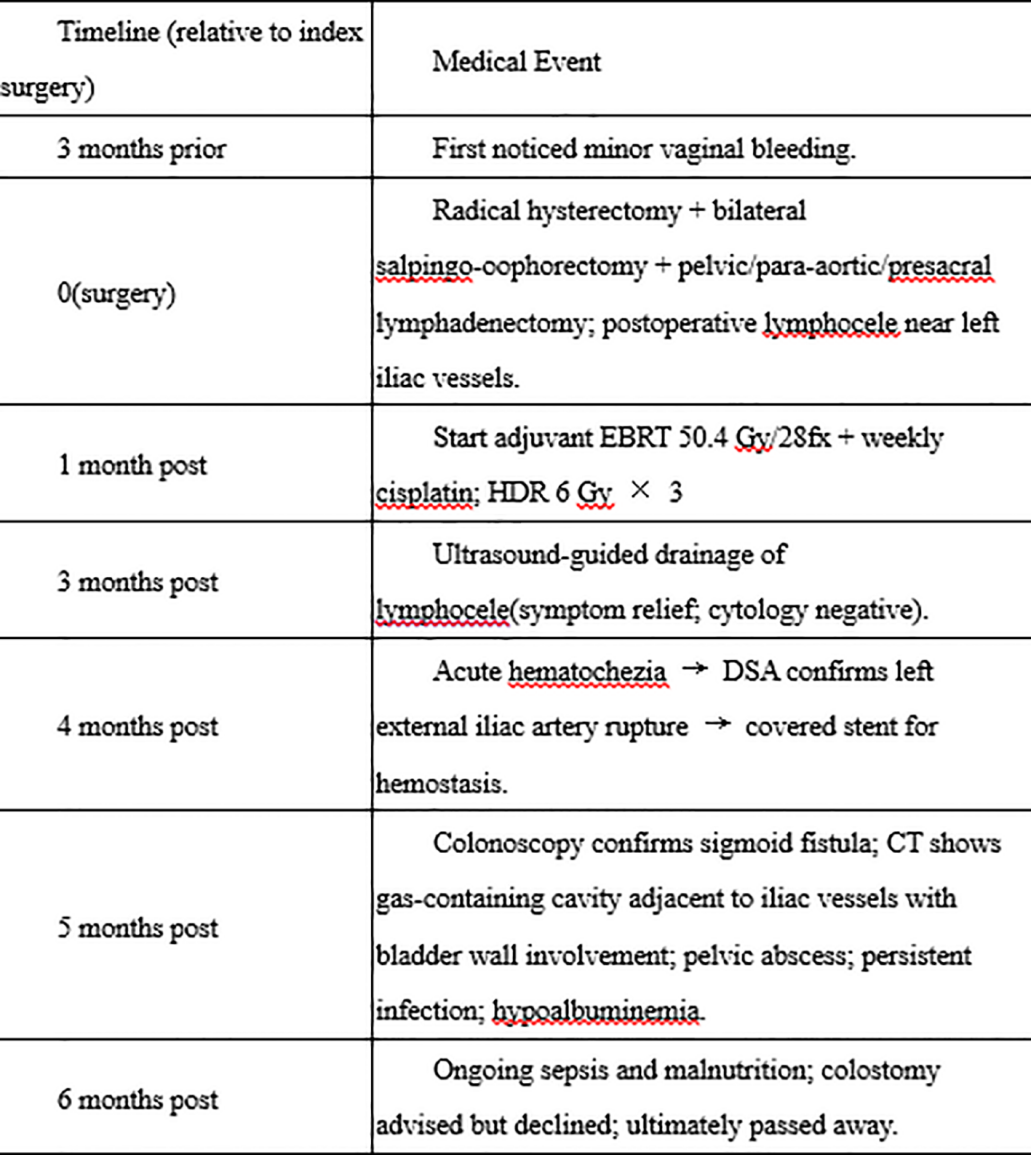
Clinical timeline of diagnosis, treatment, and complications. Schematic overview of the clinical course from symptom onset through 6 months post-surgery, including initial evaluation, radical hysterectomy, postoperative pathology (FIGO 2018 IIIC2), adjuvant chemoradiotherapy, identification and drainage of postoperative lymphocele, acute external iliac artery rupture and endovascular stent-graft placement, subsequent development of sigmoid fistula with pelvic abscess/sepsis, and transition to palliative management.

At 6 months post-surgery, she experienced multiple episodes of clinical decompensation. Given the persistent enteric fistula and uncontrolled pelvic infection, a diverting colostomy was strongly recommended to reduce fecal contamination, facilitate infection control, and prevent further catastrophic bleeding. However, the patient and her family declined surgical diversion because of concerns regarding operative risk, the anticipated poor prognosis, and the perceived impact on quality of life. Conservative management was continued, but the patient developed refractory pelvic sepsis with recurrent bleeding and progressive multi-organ dysfunction, and ultimately died. The overall diagnostic and treatment timeline is summarized in **Figure 4**.

## Discussion

Rupture of the external iliac artery is an exceptionally rare but life-threatening complication after treatment for cervical cancer. Most reported iliac arterial ruptures are associated with trauma, iatrogenic injury, or vascular intervention rather than primary oncologic therapy (21, 22). Radiation-associated vasculopathy is a recognized phenomenon; however, it is classically a delayed sequela characterized by progressive endothelial injury, fibrosis, and vessel wall fragility that may culminate in aneurysm or pseudoaneurysm formation and, rarely, rupture (10). Such events are most frequently described in the intracranial or carotid circulation after radiotherapy for brain tumors or head-and-neck malignancies, typically with a latency of 10–20 years (11, 12).In striking contrast, our patient developed rupture of the left external iliac artery and subsequently a sigmoid fistula within one month after completion of postoperative radiotherapy—an exceptionally early sequence in gynecologic oncology. Early bowel–vascular fistulae have been reported in heavily pretreated or recurrent pelvic malignancies, but early arterial rupture in the postoperative adjuvant setting remains exceedingly uncommon and generally requires concurrent precipitating factors such as infection and prior extensive surgery (20).

The unusually early onset in this case supports a multifactorial and stepwise pathogenesis rather than a single-cause mechanism. Radical hysterectomy with extensive pelvic and para-aortic lymphadenectomy can cause substantial tissue trauma and secondary fibrosis and may disrupt lymphatic channels, predisposing patients to postoperative lymphocele formation and infection. Notably, imaging obtained before adjuvant radiotherapy already demonstrated a persistent lymphocele adjacent to the left external iliac vessels, and the patient experienced progressive ipsilateral leg pain during EBRT. These findings suggest that the vascular bed was compromised primarily by postoperative factors—local compression, inflammation, and infection—before radiation exposure.

Although radiotherapy may contribute to endothelial dysfunction and impaired tissue healing, the delivered dose in this patient (EBRT 50.4 Gy plus vaginal cuff brachytherapy 6 Gy*3f) was within standard postoperative ranges. Moreover, the cumulative arterial dose was far below the historically cited threshold associated with overt arterial necrosis and rupture risk (10–12). Therefore, radiotherapy alone is unlikely to have been the dominant driver of arterial rupture. Instead, radiation likely acted as a permissive co-factor that exacerbated tissue fragility and impaired local immune and reparative capacity in a field already compromised by surgery-related lymphatic disruption, chronic inflammation, and infection. This interpretation is consistent with case evidence in gynecologic oncology in which vascular catastrophes often occur in the setting of combined insults—including surgery, recurrent disease, instrumentation, stent placement, and ongoing infection—rather than radiotherapy in isolation (15–19).

Importantly, the mechanisms underlying the two catastrophic complications in our patient were likely related but not identical. The external iliac artery rupture most plausibly resulted from postoperative vascular vulnerability compounded by a persistent and likely infected lymphocele exerting prolonged compression on the arterial wall. Continuous mechanical stress together with chronic inflammation may have progressively weakened the vessel wall, culminating in rupture. In contrast, the subsequent sigmoid fistula is more compatible with progressive pelvic sepsis and tissue breakdown within a surgically altered and irradiated pelvis. Local necrosis, abscess formation, and contamination from the gastrointestinal tract can erode adjacent bowel segments, ultimately forming a fistulous tract. In this context, the stent-graft achieved temporary hemorrhage control but could not eliminate the underlying pelvic contamination and infection, which likely contributed to recurrent bleeding and uncontrolled sepsis.

A key feature that distinguishes this case from most published reports is the upstream and potentially preventable driver: incomplete preoperative staging and a non–guideline-concordant primary surgical strategy. The absence of PET-CT and the lack of access to original MRI/CT datasets prevented accurate assessment of tumor size, stromal invasion depth, and nodal status. Consequently, the patient underwent radical hysterectomy despite ultimately having FIGO 2018 stage IIIC2 disease with extensive nodal metastasis—a scenario for which contemporary guidelines recommend definitive concurrent chemoradiation as the preferred primary treatment (8). This initial deviation likely increased the extent of lymphadenectomy, postoperative lymphatic morbidity (including lymphocele formation), susceptibility to infection, and the need for adjuvant radiotherapy, thereby creating an anatomic and biological environment vulnerable to catastrophic vascular and enteric complications. Explicit recognition of this causal chain strengthens the educational value of the report and highlights the importance of guideline-based initial management.

The literature supports the view that radiation-induced vasculopathy is typically delayed. Nanney et al. reported a mean latency of approximately 12 years between cranial radiotherapy and aneurysm detection, with over half of patients presenting with rupture or hemorrhage (11). Hanada et al. similarly described radiation-induced carotid pseudoaneurysms developing many years after nasopharyngeal irradiation (12). Conversely, early bowel–vascular fistulae in pelvic oncology are usually described in heavily pretreated or recurrent disease with extensive prior surgery and infection (20). Taken together, available evidence suggests that an early iliac arterial rupture shortly after postoperative radiotherapy should prompt clinicians to actively investigate additional contributors such as lymphocele, occult infection, or postoperative vascular injury.

This case also provides important implications for postoperative management in gynecologic oncology. First, adjuvant radiotherapy should be individualized according to postoperative recovery and risk profile rather than applied uniformly, especially in patients with significant postoperative morbidity (6). Patients with persistent lymphoceles, suspected infection, or severe pelvic symptoms before or during radiotherapy may benefit from early multidisciplinary evaluation and aggressive management of lymphatic and infectious complications before continuing adjuvant treatment. Second, in selected high-risk patients—particularly those with persistent collections adjacent to major vessels—proactive vascular imaging (CTA or MRA) during the peri-radiotherapy period may facilitate early detection of pseudoaneurysm or arterial wall compromise. Finally, once a vascular rupture or pseudoaneurysm occurs, prompt endovascular therapy (stent-graft placement or embolization) is often the preferred initial approach because of its rapidity and minimally invasive nature (18). However, when concomitant intestinal fistula or pelvic abscess is present, durable control depends on source control. Surgical diversion (e.g., colostomy) and effective drainage should be strongly considered, because conservative management frequently fails due to persistent contamination, recurrent infection, and sepsis, ultimately worsening prognosis. In our patient, refusal of diverting colostomy limited infection control and likely contributed to refractory pelvic sepsis and fatal deterioration, underscoring the critical role of shared decision-making informed by realistic risk–benefit discussions.

This report has several strengths. The temporal association of events was clearly documented, with multimodal imaging (CT, DSA, and colonoscopy) delineating the progression from lymphocele to arterial rupture and subsequent fistula formation. The case also highlights a clinically actionable and underappreciated risk pathway: inadequate baseline staging leading to non–guideline-concordant surgery, followed by complex multimodal toxicity. Nevertheless, limitations should be acknowledged. As a single case report, causality cannot be definitively established, and the inability to review original preoperative imaging limits reconstruction of the initial disease extent. In addition, not all potential vascular risk factors (e.g., pre-existing atherosclerosis or connective tissue disease) could be comprehensively evaluated. Despite these limitations, the case emphasizes the importance of comprehensive preoperative staging, guideline-based treatment selection, and vigilant postoperative surveillance to minimize the risk of catastrophic vascular and enteric complications in cervical cancer care.

## Patient perspective

The patient and her family were informed throughout the diagnostic and therapeutic process. During the final stages of her illness, the family expressed a preference for conservative management rather than surgical diversion.

## Data Availability

The raw data supporting the conclusions of this article will be made available by the authors, without undue reservation.
